# Early Factors Associated with the Development of Chronic Pain in Trauma Patients

**DOI:** 10.1155/2018/7203218

**Published:** 2018-01-30

**Authors:** Raoul Daoust, Jean Paquet, Lynne Moore, Marcel Émond, Sophie Gosselin, Gilles Lavigne, Manon Choinière, Aline Boulanger, Jean-Marc Mac-Thiong, Jean-Marc Chauny

**Affiliations:** ^1^Department of Emergency Medicine, Research Centre, Hôpital du Sacré-Coeur de Montréal, Montreal, QC, Canada; ^2^Faculté de Médecine, Université de Montréal, Montreal, QC, Canada; ^3^Research Centre, Hôpital du Sacré-Coeur (CIUSSS du Nord de-l'Île-de-Montréal), Montreal, QC, Canada; ^4^Département de médecine sociale et préventive, Faculté de médecine, Université Laval, Quebec, Canada; ^5^Axe de recherche en traumatologie-urgence-soins intensifs du Centre de recherche FRQS du CHU-Québec, Quebec, Canada; ^6^Département de médecine familiale et de médecine d'urgence, Faculté de médecine, Université Laval, Quebec, Canada; ^7^Department of Emergency Medicine, McGill University Health Centre, McGill University, Montreal, QC, Canada; ^8^Faculties of Dental Medicine and Medicine, Université de Montréal, Montreal, QC, Canada; ^9^Center for Advanced Research in Sleep Medicine, Hôpital du Sacré-Coeur de Montréal (CIUSSS du Nord de-l'Île-de-Montréal), Montreal, QC, Canada; ^10^Centre de recherche du Centre Hospitalier de l'Université de Montréal (CRCHUM), Montreal, QC, Canada; ^11^Département d'anesthésiologie, Faculté de médecine, Université de Montréal, Montreal, QC, Canada

## Abstract

**Objective:**

To identify factors, available at the time of trauma admission, associated with the development of chronic pain to allow testing of preventive approaches.

**Methods:**

In a retrospective observational cohort study, we included all patients ≥ 18 years old admitted for injury in 57 adult trauma centers in the province of Quebec (Canada) between 2004 and 2014. Chronic pain was defined as follows: treated in a chronic pain clinic, diagnosed with chronic pain, or received at least 2 prescriptions of chronic pain medications 3 to 12 months postinjury.

**Results:**

A total of 95,134 patients were retained for analysis. Mean age was 59.8 years (±21.7), and 52% were men. The causes of trauma were falls (63%) and motor vehicle accidents (22%). We identified 14,518 patients (15.3%; 95% CI: 15.1–15.5) who developed chronic pain. After controlling for confounding factors, the variables associated with chronic pain were spinal cord injury (OR = 3.9; 95% CI: 3.4–4.6), disc-vertebra trauma (OR = 1.6; 95% CI: 1.5–1.7), history of alcoholism (OR = 1.4; 95% CI: 1.2–1.7), history of anxiety (OR = 1.4; 95% CI: 1.2–1.5), history of depression (OR = 1.3; 95% CI: 1.1–1.4), and being female (OR = 1.3; 95% CI: 1.2–1.3). The area under the receiving operating characteristic curve derived from the model was 0.80.

**Conclusions:**

We identified risk factors present on hospital admission that can predict trauma patients who will develop chronic pain. These factors should be prospectively validated.

## 1. Introduction

Traumatic injury accounts for approximately 37 million emergency department visits each year in the US [1]. For many, the injury will resolve without complications, but for others, a heightened reactivity of the nervous system called central sensitization [2, 3] will trigger persistent pain long after the traumatic event. Thus, a proportion of trauma patients will eventually develop chronic pain [4], which is commonly defined as ongoing pain experienced on most days lasting for at least 3 months [5].

The prevalence of posttraumatic chronic pain varies greatly between and within injury types: from 22 to 93% in orthopedic trauma [6], 26–96% for spinal cord injuries [7], and 40–75% for traumatic brain injuries [7]. Significant variability in the prevalence of chronic pain also exists in postsurgical studies (5–85%) and depends largely on the type of surgery [8]. Variability in the prevalence of posttraumatic chronic pain can be partially explained by the type of injury and surgery performed, characteristics of the study population, heterogeneity of pain outcome measures [4], and definition of a chronic pain patient [9]. To define chronic pain, studies used different validated measures of pain intensity, presence/absence of pain, and pain questionnaires at various time points after the injury [6].

Several studies have identified risk factors of chronic pain development in trauma patients [4, 7]. Demographic, injury-related, and psychological factors have been shown to contribute to the transition from acute to chronic pain in trauma patients [10]. Being female, older age (≥65), fewer years of education, injury severity, high pain intensity during hospitalization and at hospital discharge, preinjury alcohol use disorder, anxiety or depression, postinjury anxiety, depression or PTSD, and eligibility for compensation have been associated with the development of chronic pain in trauma patients [6, 7, 10–12]. Some of these factors are present at hospital admission (sociodemographics, injury details, and history of anxiety, depression, or alcoholism), some are available during hospitalization (pain intensity during hospital stay and at discharge, type of surgery, and intensive care duration), while others are identified after hospitalization (anxiety, depression, PTSD symptoms, and pain catastrophizing).

If used during the acute pain phase, some therapeutic strategies (behavioural, cognitive, and drug approaches) may help prevent the development of chronic pain [7]. As primary prevention strategies, these treatments must be administered before the occurrence of the pain chronicization process, justifying the need to find factors present at hospital admission that can identify patients at risk of developing chronic pain [7].

The main objective of the present study was to identify factors, available at hospital admission, associated with the development of chronic pain for trauma patients and to develop a predictive model.

## 2. Methods

### 2.1. Study Design and Population

A retrospective multicenter cohort study was conducted using three government population databases. All patients of 18 years and older admitted for injury to any one of 57 adult trauma centers (3 level I, 5 level II, 21 level III, and 28 level IV trauma centers) in the province of Quebec (Canada) between 2004 and 2014 were included in the study. Patients who died or with a follow-up period less than 1 year and those with multiple trauma episodes (difficult to identify which trauma episode was associated with chronic pain) were excluded.

### 2.2. Study Databases

The *Quebec Trauma Registry* was developed in 1993 and involves mandatory data collection for patients admitted to any provincial trauma center according to the following inclusion criteria: death following injury, hospital stay > 2 days, intensive care unit admission, or transfer from another hospital. Medical archivists extract registry data from patients' medical files, using standardized coding protocols. Anatomic injuries are coded with the Abbreviated Injury Scale (AIS) according to guidelines published by the Association for the Advancement of Automotive Medicine [13]. The registry is centralized at the Régie de l'assurance maladie du Québec of the Quebec Ministry of Health and is subject to periodic validation.

*MED-ECHO* (*Maintenance et exploitation des données pour l'étude de la clientèle hospitalière*) is a medico administrative database managed by the Quebec Ministry of Health. It contains information on principal and secondary diagnoses and medical interventions for all hospitalizations in the province of Quebec. For each included patient, access to MED-ECHO information was granted for a time period ranging from 1 year before to 8 years after the target injury.

The *RAMQ* medical consultations and medication database of the Régie de l'Assurance Maladie du Québec is an administrative database maintained by the Quebec Ministry of Health and contains diagnostic information and specific codes for chronic pain visits for all medical consultations in the province of Quebec. It also contains information on all medication prescriptions filled for Quebec residents covered by the Quebec prescription drug insurance plan, which represent approximately 50% of all included individuals. The RAMQ database provided information on included patients for the same time period as for the MED-ECHO database.

The three databases were linked using a unique anonymous identification number provided by the “Régie de l'assurance maladie du Québec.” Access to these databases required the ethic approval of the “Commission d'accès à l'information du Québec” (CAI) and the “Responsable de l'accès à l'information et de la protection des renseignements personnels de la RAMQ” (RAI-RAMQ). The CAI and the RAI-RAMQ approved our study for cases registered in the Quebec Trauma Registry between 2004 and 2014.

### 2.3. Main Outcome

Chronic pain (pain experienced for at least 3 months) patients were identified using any of the following 3 criteria: (1) Patients who were referred to a specialized chronic pain clinic (chronic pain center special code from the RAMQ database). (2) Patients diagnosed with chronic pain (hospitalization diagnosis derived from the MED-ECHO or RAMQ database) during their entire follow-up period (up to 8 years after injury). We used the entire follow-up (from 3 months to 8 years after injury) for these chronic pain criteria since access to specialized chronic pain clinics can extend for years. (3) Patients who filled at least 2 prescriptions of opioids or at least 1 prescription of chronic pain medication [7] (amitriptyline, gabapentin, or pregabalin from the RAMQ database) from 3 to 12 months post injury. Patients who already presented one of these three chronic pain defining criteria during the 1-year period preceding the target injury were excluded from data analysis.

### 2.4. Database Variables

From the three databases, we extracted patient characteristics, types of trauma, and available factors associated with chronic pain identified in the literature: age, sex, injury mechanisms (fall, motor accident, weapon or blunt object, and other), injury severity score (ISS), abbreviated injury scale (AIS), emergency department (ED) stay duration, and history of alcoholism, depression, or anxiety 1 year prior to target injury. A score greater than 15 on the ISS was used to define major trauma or polytrauma [14]. The first two digits of the AIS code were used to identify the injury and regions of each wound (example: AIS code of 852000.2: foot fracture was recoded into lower extremity/skeletal). One-year preinjury history of alcoholism (alcohol dependence syndrome, acute alcoholic hepatitis, alcohol-induced mental disorders, and alcoholic gastritis), depression (prolonged depressive reaction, dysthymic disorder, and depressive disorder), and anxiety (anxiety states, phobic disorders, and obsessive-compulsive disorders) was extracted from ICD-9 diagnoses included in RAMQ medical consultations or MED-ECHO hospitalization databases.

### 2.5. Statistical Analyses

We used univariate statistics (Chi-square and *t*-tests) to compare the characteristics of the included patients versus those who were excluded (died or had a follow-up less than 1 year). We randomly selected two-thirds of cases to generate a derivation sample used to create a model to predict chronic pain and the last third was used as a validation sample [15]. We used univariate statistics (Chi-square and *t*-tests) to compare the characteristics of the derivation and the validation samples. Since small differences can result in a statistically significant test in very large samples, Cohen's effect sizes are presented instead of *p* values. Small, medium, and large effect sizes for Chi-square are 0.1, 0.3, and 0.5, respectively, and for the *t*-test statistic, 0.2, 0.5, and 0.8, respectively [16].

On the derivation sample, univariate logistic regressions were used to compare the chronic pain predictors of patients with or without chronic pain criteria. Predictors with significant odd ratios and those identified in the literature (age, sex, injury mechanisms, major trauma, and history of alcoholism, depression, or anxiety) were selected for multivariate analysis. We used a multivariate logistic regression analysis with backward stepwise procedure to develop the model on the derivation sample using chronic pain development as the dependent variable. Performance of the derivation sample model was assessed using Nagelkerke's *R*^2^, which is the proportion of variance explained by the predictors on the dependent variable and the Brier score which measures the accuracy of probabilistic predictions. The Brier score can range from 0 for a perfect model to 0.25 for an uninformative model. The Hosmer–Lemeshow test was used to evaluate the calibration of the derivation model. A statistically nonsignificant result suggests good agreement between predicted and observed probabilities. Finally, the discrimination of the derivation model was assessed with the c-statistic, which represents the area under the ROC curve.

On the validation sample, the logistic regression equation (derived on the derivation sample) was used to predict chronic pain. Performance and discrimination were also evaluated using the Brier score and the c-statistic, respectively.

Alpha levels were set at 0.05 and all analyses were performed using SPSS version 23 (IBM, Somers, NY). Results are reported according to the “Transparent Reporting of a multivariable prediction model for Individual Prognosis Or Diagnosis” (TRIPOD) Statement [17].

## 3. Results

**Figure 1:**
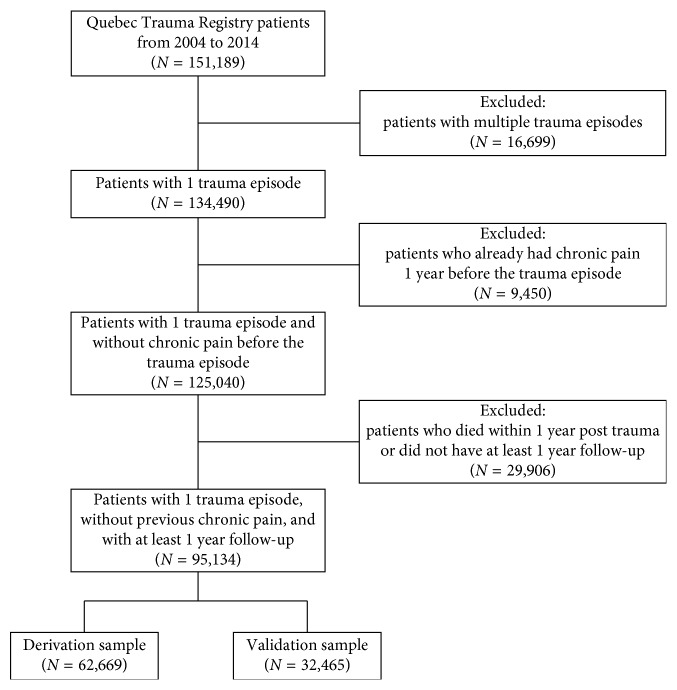
Flow chart of patients' study inclusion.

The Québec Trauma Registry included a total of 151,189 adult patients admitted for an injury between January 1, 2004, and March 31, 2014. Of these patients, 11% were excluded for having more than one injury episode, 6.3% because they met the chronic pain criteria during the year preceding their target injury episode, and 19.8% because they died or had a follow-up shorter than a year. A total of 95,134 subjects to create the derivation and validation sample were retained (Figure 1).  Table 1 shows the characteristics of the patients included in the study and those excluded. Excluded patients were similar in all aspects to the selected sample except for having more falls and for being older. This was expected since patients who died within a year of the trauma episode were generally older.

The mean age of the selected sample was 59.8 years (SD ± 21.7), almost half (48.1%) were female, and the mean follow-up duration was 4.8 years (SD ± 2.4). The most common mechanism of injury was falls (63%), 17% had major trauma, and the average time spent in the ED was 17 hours. In the whole sample, 14,518 patients (15.3%; 95% CI: 15.1–15.5) developed chronic pain during the follow-up period according to our three criteria: 92% of the chronic pain patients were identified through chronic pain medication consumption, 7% had a chronic pain clinic consultation code, and 4% were diagnosed with chronic pain when subsequently hospitalized.

Univariate comparisons of patients' characteristics for the derivation and the validation samples are presented in Table 2. No significant differences were observed between the two samples.  Table 3 shows the comparison of hospital admission variables for patients with or without chronic pain in the derivation sample. Age, sex, injury mechanisms, injury regions (brain-brainstem-cerebellum, disc-vertebra, thorax/skeletal, face/skeletal, abdomen/organ, spinal cord, face/whole area, skull fracture, thorax/organ, face/organ, upper extremity/whole area, or loss of consciousness), ED stay duration, and history of alcoholism, depression, or anxiety a year prior to target injury were all significantly associated with chronic pain development. Spinal cord injury was the most important predictor of chronic pain development.

Because collinearity was strong between insurance status and age (patients aged 65 or older were almost all covered by the Quebec prescription drug insurance plan), age was removed from the multivariate analysis. Results of the multivariate logistic regression analysis using the derivation sample (*N* = 62,669) are presented in Table 4. After controlling for the Quebec prescription drug insurance plan status and follow-up duration, the following factors were significantly associated with the development of chronic pain: being female, spinal cord damage, disc-vertebra injury, thorax-skeletal injury, loss of consciousness, and history of alcoholism, depression, or anxiety in the year prior to target injury. Having a weapon or blunt object wound (compared to a fall) and a brain-brainstem-cerebellum injury was associated with less development of chronic pain. When controlled for all other variables, spinal cord injury was still the most important predictor of chronic pain. The Hosmer–Lemeshow *p* value calculated on the derivation sample was 0.01, the Nagelkerke's percentage of variance explained was 0.28, the Brier score was 0.11, and area under the ROC curve was 0.80 (95% CI: 0.79–0.81). Except for calibration (Hosmer–Lemeshow), performance (Nagelkerke's and Brier score) and discrimination (area under the ROC curve) values are within the range of an informative model.

When the logistic regression equation using the same predictors was applied to the validation sample (*N* = 32,465), the Brier score remained at 0.11 and the area under the ROC curve was estimated at 0.80 (95% CI: 0.80–0.81) which is consistent with strong discrimination [18].

In a sensitivity analysis on the derivation sample, we performed the same stepwise logistic regression using only patients covered by the Quebec prescription drug insurance plan (*N* = 31,715). Except for loss of consciousness, which was no longer significant, the same set of predictors with approximately the same odds ratios was associated with the development of chronic pain.

## 4. Discussion

This study has shown that 10 risk factors present on hospital admission can reliably predict trauma patients who will develop chronic pain. The chronic pain development predictors of our final model were similar to those found in previous studies. Sex (being female), injury characteristics, and history of alcoholism, anxiety, or depression have all previously been identified as predictors of chronic pain [6, 10–12, 19–22]. Patients with two or more rib fractures, patients with sternal fractures, and patients with mild traumatic brain injury were also reported at risk of chronic pain [23–25]. The most important predictor of chronic pain development found in our study was spinal cord injury with an odds ratio of 3.9. Back and spine problems have frequently been identified as a major predictor of the transition from acute to chronic pain [26, 27].

However, our study was done on a very large sample size (95,134 patients) from the Quebec Trauma Registry, a reliable government supervised database. For example, inclusion in the Quebec Trauma Registry is mandatory; it uses standardized coding protocols and is subject to periodic validation. Our study is also the first to focus on risk factors present on hospital admission. Furthermore, we validated our predictive model on a large distinct sample (32,465 patients). This predictive model could allow for the prompt screening of the trauma population at risk of developing chronic pain, early testing, and implementation of preventive approaches.

The discrimination power of our model is good (c-statistic = 0.80 with the validation sample) and is higher than a recently published study predicting chronic pain development in patients with acute low back pain [27]. However, our discrimination level suggests that other predictors not available in the trauma registry database or factors occurring after admission also contribute to the development of chronic pain. Level of education, pain intensity at hospital admission, and eligibility for disability compensation are factors that could potentially increase the discriminative power of our model, and their impact should be studied prospectively. Furthermore, individual factors such as properties of the brain's emotional learning circuitry [28], corticotropin-releasing hormone binding protein (CRHBP) gene polymorphisms [29], and brain's white matter structural properties [30] have been recently proposed as predictors of development of chronic pain but infer more invasive and costly investigations.

The prevalence of chronic pain development found in the present study (15.3%) is lower than that observed in two major reviews in trauma populations (from 22% to 96%) [6, 7]. The chronic pain definition used in the present study could explain this discrepancy. Only 7% of the patients who were recognized as chronic pain patients in our study were patients consulting specialized chronic pain clinics. Either the code related to consultation at a specialized chronic pain clinic was not systematically entered in the databases or accessibility to these clinics is restrained [31]. In both cases, the prevalence of chronic pain development may have been underestimated.

This study has other limitations. The majority (92%) of our chronic pain patients were identified through the use of the chronic pain medication criteria. Since almost half of our trauma population were not covered by the Quebec prescription drug insurance plan, the prevalence of chronic pain development was likely underestimated, even in the presence of the other two criteria.

The definition of chronic pain in our study may introduce other biases in the estimation of chronic pain prevalence and could also affect our risk factor modelling. Since we used the following proxy—filling at least 2 pain medication prescriptions within 3 to 12 months after the target injury—to define chronic pain development, we cannot exclude that some patients filled their prescriptions but did not consume any medication, or were taking pain medication for a subsequent injury/health problem or even became addicted to the pain medication. The retrospective design of this study limited our choice of chronic pain development predictors to variables included in the Quebec Trauma Registry. For example, pain intensity level on hospital admission, which has been shown as a consistent predictor of chronic pain in a trauma population, was not available in the Quebec Trauma Registry. Finally, history of alcoholism, depression, and anxiety was limited to the year preceding the target injury. Prospective studies are needed to evaluate and improve the ability of our model to predict chronic pain development from predictors that are available at hospital admission.

## 5. Conclusions

Despite the relatively low incidence of chronic pain in our injury cohort, we identified risk factors present on hospital admission that can reliably predict trauma patients who will develop chronic pain. These factors should be prospectively validated. Hospital admission screening of the trauma population at risk of developing chronic pain could allow for early testing and implementation of preventive approaches.

## Figures and Tables

**Table 1:** Characteristics of included and excluded patients.

Characteristics	Included patients (*N* = 95,134)	Excluded patients (*N* = 29,906)	ES
Age (%) ≥ 65 years	45.2	75.3	0.26
Female (%)	48.1	54.2	0.05
Mechanism of injury (%)			
Fall	63.3	80.8	0.16
Motor vehicle accident	21.5	10.2	—
Weapon or blunt object	8.3	4.1	—
Other	7.0	4.9	—
Major trauma (ISS > 15) (%)	17.0	17.5	0.01
History of alcoholism (%)	1.6	2.0	0.01
History of depression disorder (%)	5.1	5.4	<0.01
History of anxiety disorder (%)	6.8	6.8	<0.01
Mean (±SD) ED stay duration (hrs)	17.0 (18.0)	19.6 (19.7)	0.14^∗^

ES: effect size from the chi-square test; ^∗^effect size from the *t*-test. Small, medium, and large effect sizes for chi-square are 0.1, 0.3, and 0.5, respectively, and for the *t*-test statistic 0.2, 0.5, and 0.8, respectively.

**Table 2:** Univariate comparisons of patients' characteristics in the derivation and validation samples.

Characteristics	Derivation sample (*N* = 62,669)	Validation sample (*N* = 32,465)	ES
Age (%) ≥ 65	45.4	44.9	<0.01
Female (%)	48.1	48.2	<0.01
Mechanism of injury (%)			
Fall	63.5	62.8	<0.01
Motor vehicle accident	21.3	21.8	—
Weapon or blunt object	8.2	8.4	—
Other	7.0	7.0	—
Major trauma (ISS > 15) (%)	17.1	16.9	<0.01
History of alcoholism (%)	1.6	1.6	<0.01
History of depression disorder (%)	5.0	5.2	<0.01
History of anxiety disorder (%)	6.8	6.9	<0.01
With Quebec medication insurance (%)	50.6	50.0	<0.01
Mean (±SD) follow-up (years)	4.8 (2.4)	4.8 (2.4)	<0.01^∗^
Mean (±SD) ED stay duration (hrs)	17.0 (18.2)	16.9 (17.7)	<0.01^∗^
Posttrauma chronic pain (%)	15.4	15.1	<0.01

ES: effect size from the chi-square test; ^∗^effect size from the *t*-test. Small, medium, and large effect sizes for chi-square are 0.1, 0.3, and 0.5, respectively, and for the *t*-test statistic 0.2, 0.5, and 0.8, respectively.

**Table 3:** Univariate comparisons of variables for patients with and without chronic pain in the derivation sample.

Variables	Without chronic pain (*N* = 53,040)	With chronic pain (*N* = 9,629)	Odd ratio (95% CI)
Age (%) ≥ 65	42.6	60.4	**2.05 (1.96–2.14)**
Female (%)	46.6	56.5	**1.49 (1.42–1.55)**
Mechanism of injury (%)			
Fall	62.7	68.0	Reference
Motor vehicle accident	21.5	20.2	**0.87 (0.82–0.91)**
Weapon or blunt object	8.7	5.5	**0.59 (0.53–0.64)**
Other	7.1	6.3	**0.86 (0.76–0.91)**
AIS injury regions (%)			
Lower extremity/skeletal	52.4	53.3	1.04 (1.00–1.08)
Upper extremity/skeletal	21.5	21.3	1.00 (0.94–1.04)
Thorax/skeletal (rib or sternum)	12.5	14.7	**1.20 (1.13–1.28)**
Disc, vertebra	11.2	14.9	**1.39 (1.31–1.48)**
Lower extremity/whole area^a^	10.7	10.9	1.03 (0.96–1.10)
Face/whole area^b^	10.7	9.6	**0.88 (0.82–0.95)**
Upper extremity/whole area^a^	10.1	9.4	**0.92 (0.85–0.99)**
Brain, brainstem, cerebellum	9.9	8.1	**0.80 (0.74–0.86)**
Head/whole area^c^	8.9	8.9	1.00 (0.92–1.08)
Face/skeletal	7.0	5.5	**0.77 (0.70–0.84)**
Thorax/organ^d^	5.8	5.1	**0.87 (0.79–0.95)**
Abdomen/organ^e^	5.4	4.5	**0.82 (0.74–0.91)**
Skull fracture	4.6	3.2	**0.69 (0.61–0.78)**
Loss of consciousness	4.4	5.1	**1.17 (1.06–1.30)**
Thorax/whole area^f^	3.3	3.4	1.03 (0.91–1.16)
Lower extremity/MTL^g^	3.1	2.7	0.88 (0.77–1.00)
Abdomen/whole area^h^	2.7	2.8	1.04 (0.91–1.19)
Face/organ^i^	2.5	2.0	**0.78 (0.67–0.91)**
Upper extremity/MTL^g^	2.3	2.2	0.94 (0.81–1.08)
Spinal cord	2.1	5.0	**2.51 (2.25–2.80)**
Major trauma (ISS > 15) (%)	17.0	17.5	1.03 (0.98–1.10)
History of alcoholism (%)	1.5	2.3	**1.59 (1.37–1.85)**
History of depression disorder (%)	4.9	6.0	**1.25 (1.14–1.37)**
History of anxiety disorder (%)	6.4	9.2	**1.48 (1.37–1.60)**
Mean (±SD) follow-up duration (yr)	4.7 (2.4)	5.0 (2.3)	**1.06 (1.05–1.07)**
Mean (±SD) ED stay duration (hrs)	16.8 (18.1)	18.3 (19.0)	**1.01 (1.00–1.01)**

a: amputation, crushing injury, penetrating trauma, contusion, or laceration; b: superficial penetrating trauma, contusion, or laceration; c: penetrating trauma, contusion, or laceration; d: air way, lung, diaphragm, oesophagus, or heart; e: perineal, scrotum, penis, vagina, adrenal, bladder, bowel, liver, and kidney; f: crushing injury, penetrating trauma, laceration, or contusion; g: muscle, tendon, and ligament; h: superficial penetrating trauma, contusion, or laceration; i: eye, ear, or mouth. Odd ratios in bold were significant at *p* < 0.05.

**Table 4:** Results of the multivariate logistic regression analysis to predict the development of chronic pain in the derivation sample (*N* = 62,669).

Predictors^∗^	Odd ratios	95% CI
Female	**1.26**	**1.19–1.33**
Mechanism of injury		
Fall	Reference	—
Motor vehicle accident	1.06	0.99–1.14
Weapon or blunt object	**0.87**	**0.78–0.98**
Other	**1.12**	**1.00–1.25**
AIS injury regions		
Spinal cord	**3.94**	**3.40–4.55**
Disc, vertebra	**1.58**	**1.46–1.70**
Thorax/skeletal (rib or sternum)	**1.19**	**1.10–1.28**
Loss of consciousness	**1.19**	**1.05–1.34**
Brain, brainstem, cerebellum	**0.83**	**0.76–0.91**
History of alcoholism (%)	**1.41**	**1.18–1.69**
History of depression (%)	**1.26**	**1.13–1.41**
History of anxiety (%)	**1.36**	**1.24–1.49**

^∗^Because of strong collinearity between insurance status and age (patients aged ≥  65 were almost all covered by the Quebec prescription drug insurance plan), age was removed from the multivariate analysis. Odd ratios in bold were significant at *p* < 0.05.

## References

[1] National Trauma Institute (March 2017). The case for funding trauma research. http://www.nationaltraumainstitute.net/pdf/case_trauma_funding-old.pdf.

[2] Latremoliere A., Woolf C. J. (2009). Central sensitization: a generator of pain hypersensitivity by central neural plasticity. *Journal of Pain*.

[3] Arendt-Nielsen L., Graven-Nielsen T. (2003). Central sensitization in fibromyalgia and other musculoskeletal disorders. *Current Pain and Headache Reports*.

[4] Berube M., Choiniere M., Laflamme Y. G., Gélinas C. (2016). Acute to chronic pain transition in extremity trauma: a narrative review for future preventive interventions (part 1). *International Journal of Orthopaedic and Trauma Nursing*.

[5] Treede R. D., Rief W., Barke A. (2015). A classification of chronic pain for ICD-11. *Pain*.

[6] Rosenbloom B. N., Khan S., McCartney C., Katz J. (2013). Systematic review of persistent pain and psychological outcomes following traumatic musculoskeletal injury. *Journal of Pain Research*.

[7] Radresa O., Chauny J. M., Lavigne G., Piette E., Paquet J., Daoust R. (2014). Current views on acute to chronic pain transition in post-traumatic patients: risk factors and potential for pre-emptive treatments. *Journal of Trauma and Acute Care Surgery*.

[8] Macrae W. A. (2008). Chronic post-surgical pain: 10 years on. *British Journal of Anaesthesia*.

[9] Rosenbloom B. N., Katz J., Chin K. Y. (2016). Predicting pain outcomes after traumatic musculoskeletal injury. *Pain*.

[10] Berube M., Choiniere M., Laflamme Y. G., Gélinas C. (2017). Acute to chronic pain transition in extremity trauma: a narrative review for future preventive interventions (part 2). *International Journal of Orthopaedic and Trauma Nursing*.

[11] Holmes A., Williamson O., Hogg M. (2010). Predictors of pain severity 3 months after serious injury. *Pain Medicine*.

[12] Rivara F. P., Mackenzie E. J., Jurkovich G. J., Nathens A. B., Wang J., Scharfstein D. O. (2008). Prevalence of pain in patients 1 year after major trauma. *Archives of Surgery*.

[13] Association for the Advancement of Automotive Medicine (1990). *Abbreviated Injury Scale (AIS)*.

[14] Copes W. S., Champion H. R., Sacco W. J., Lawnick M. M., Keast S. L., Bain L. W. (1988). The Injury Severity Score revisited. *Journal of Trauma: Injury, Infection, and Critical Care*.

[15] Steyerberg E. W. (2009). *Clinical Prediction Models: A Practical Approach to Development, Validation, and Updating*.

[16] Cohen J. (1988). *Statistical Power Analysis for the Behavioral Sciences*.

[17] Moons K. G., Altman D. G., Reitsma J. B. (2015). Transparent Reporting of a multivariable prediction model for Individual Prognosis or Diagnosis (TRIPOD): explanation and elaboration. *Annals of Internal Medicine*.

[18] Hosmer D. W., Lemeshow S. (2000). *Applied Logistic Regression*.

[19] Clay F. J., Watson W. L., Newstead S. V., McClure R. J. (2012). A systematic review of early prognostic factors for persisting pain following acute orthopedic trauma. *Pain Research and Management*.

[20] Castillo R. C., MacKenzie E. J., Wegener S. T., Bosse M. J. (2006). Prevalence of chronic pain seven years following limb threatening lower extremity trauma. *Pain*.

[21] Holmes A., Williamson O., Hogg M., Arnold C., O’Donnell M. L. (2013). Determinants of chronic pain 3 years after moderate or serious injury. *Pain Medicine*.

[22] Holmes A., Williamson O., Hogg M. (2010). Predictors of pain 12 months after serious injury. *Pain Medicine*.

[23] Daoust R., Emond M., Bergeron E. (2013). Risk factors of significant pain syndrome 90 days after minor thoracic injury: trajectory analysis. *Academic Emergency Medicine*.

[24] Nampiaparampil D. E. (2008). Prevalence of chronic pain after traumatic brain injury: a systematic review. *JAMA*.

[25] Mayberry J. C., Kroeker A. D., Ham L. B., Mullins R. J., Trunkey D. D. (2009). Long-term morbidity, pain, and disability after repair of severe chest wall injuries. *American Surgeon*.

[26] Mehling W. E., Ebell M. H., Avins A. L., Hecht F. M. (2015). Clinical decision rule for primary care patient with acute low back pain at risk of developing chronic pain. *Spine Journal*.

[27] Traeger A. C., Henschke N., Hubscher M. (2016). Estimating the risk of chronic pain: development and validation of a prognostic model (PICKUP) for patients with acute low back pain. *PLoS Medicine*.

[28] Apkarian A. V., Baliki M. N., Farmer M. A. (2013). Predicting transition to chronic pain. *Current Opinion in Neurology*.

[29] Linnstaedt S. D., Bortsov A. V., Soward A. C. (2016). CRHBP polymorphisms predict chronic pain development following motor vehicle collision. *Pain*.

[30] Mansour A. R., Baliki M. N., Huang L. (2013). Brain white matter structural properties predict transition to chronic pain. *Pain*.

[31] Lynch M. E., Campbell F. A., Clark A. J. (2007). Waiting for treatment for chronic pain—a survey of existing benchmarks: toward establishing evidence-based benchmarks for medically acceptable waiting times. *Pain Research and Management*.

